# Reliability and validity of a quick test of cognitive speed (AQT) in screening for mild cognitive impairment and dementia

**DOI:** 10.1186/s12877-021-02621-z

**Published:** 2021-12-15

**Authors:** Pouya Farokhnezhad Afshar, Elisabeth H. Wiig, Seyed Kazem Malakouti, Behnam Shariati, Sara Nejati

**Affiliations:** 1 School of Behavioral Sciences and Mental Health (Tehran Institute of Psychiatry), Iran University of Medical Sciences, Shahid Mansouri Street, Niyayesh Street, Satarkhan Avenue, Tehran, 1445613111 Iran; 2 Department of Communication Sciences and Disorders and Knowledge Research Institute, Boston University, Inc., 2131 Reflection Bay Drive, Arlington, TX 76013 USA; 3 Mental Health Research Center, Iran University of Medical Sciences, Tehran, Iran; 4 Iranian Research Center on ageing, University of Social Welfare and Rehabilitation Sciences, Tehran, Iran

**Keywords:** Aged, Cognition disorders, Mental status, Dementia tests

## Abstract

**Background:**

Cognitive disorders are one of the important issues in old age. There are many cognitive tests, but some variables affect their results (e.g., age and education). This study aimed to evaluate the reliability and validity of A Quick Test of Cognitive Speed (AQT) in screening for mild cognitive impairment (MCI) and dementia.

**Methods:**

This is a psychometric properties study. 115 older adults participated in the study and were divided into three groups (46 with MCI, 24 with dementia, and 45 control) based on the diagnosis of two geriatric psychiatrists. Participants were assessed by AQT and Mini-Mental State Examination (MMSE). Data were analyzed using Pearson correlation, independent t-test, and ROC curve by SPSS v.23.

**Results:**

There was no significant correlation between AQT subscales and age and no significant difference between the AQT subscales in sex, educational levels. The test-retest correlations ranges were 0.84 from 097. Concurrent validity was significant between MMSE and AQT. Its correlation was with Color − 0.78, Form − 0.71, and Color-Form − 0.72. The cut-off point for Color was 43.50 s, Form 52 s, and Color-Form 89 s were based on sensitivity and specificity for differentiating older patients with MCI with controls. The cut-off point for Color was 62.50 s, for Form 111 s, and Color-Form 197.50 s based on sensitivity and specificity measures for differentiating older patients with dementia and MCI.

**Conclusion:**

The findings showed that AQT is a suitable tool for screening cognitive function in older adults.

## Background

An increase in the aging population is associated with a higher prevalence of diseases and syndromes in older people, one of which is cognitive disorders. Cognitive impairment is a common neurological disorder in old age and includes a wide range of conditions from mild cognitive impairment (MCI) to advanced dementia [1]. MCI is a precursor to dementia and is a transition from age-related cognitive decline to more severe cognitive disorders [2]. Different mechanisms such as amyloid deposition, inflammation, an increase in free radicals, loss of synapses and neurons, and dysfunction of neurotransmitters lead to dementia [1]. There are about 50 million people in the world with dementia, and ten million new cases are added each year. It is estimated that the number of people with neurocognitive disorder (NCD) will reach 152 million by 2050 [3].

The definitive diagnosis of dementia is possible only by histopathological examination of brain tissue after death, therefore, most cases are diagnosed based on clinical information [4]. Clinical paradigms that assess cognitive function range from short memory tests to comprehensive assessment scales [5]. Cognitive disorders are generally assessed by broader neuropsychological tests [6]. Traditional cognitive status scales show little sensitivity in distinguishing between the normal range of cognitive function and cognitive impairments [5] and are influenced by culture, language, and education [7]. The Mini-Mental State Examination (MMSE) and the Clock Drawing Test (CDT) are diagnostic tests for dementia, whose accuracy is still questionable, because their score changes with age [8, 9], and which limits the ability to diagnose patients with early-stage dementia and MCI [10]. It is stated that 3.46% of the MMSE standard deviation is related to age and education [11]. Some studies have even suggested that these tools be used together for more accuracy [12, 13]. Montreal Cognitive Assessment (MoCA) is also a tool for screening for MCI, It has been reported to have a sensitivity range of about 86 to 95% [14, 15]. But all these tools require a minimum of literacy (such as calculating, reading, and writing).

In contrast, the use of visual-verbal scales such as A Quick Test of Cognitive Speed (AQT) that are not influenced by factors such as gender, formal education beyond the acquisition of literacy (Grades 5 to 8), and culture, can distinguish between normal aging and cognitive disorders caused by disease [16].

The processing speed theory of adult age states that the decrease in processing speed is due to cognitive decline, not to the reduction in or lack of information [17]. Rapid Automatized Naming (RAN) is the ability to perceive a visual symbol such as letter, Color, and Form, or retrieve it quickly and accurately. Stroop in 1953 designed the first RAN test, the Stroop Color, and Word Test. This test involves, among others, the ability to consistently read the names of colors printed in contrasting colors, thus inhibiting responses to distracting features. Denckla and Rudel in 1976 used continuous naming of numbers, shapes, letters, and colors to evaluate RAN speed. Wiig (1984) designed a Color (C), Form (F), and Color-Form (CF) processing-speed test to probe RAN abilities in children with language disorders [18]. A Quick Test of Cognitive Speed (AQT) was later designed by Wiig et al. to compare processing speed in adults with clinical diagnoses of dementia and neurotypical age peers [18–21]. AQT is a visual-verbal processing speed test that evaluates aspects of executive function and can be used in a variety of languages and cultures [5, 16, 22]. AQT measures the speed of perception, retrieval, and naming of basic colors and forms in single-dimension naming and cognitive speed associated with central executive functions (attention, working memory, and set shifting) in dual-dimension naming of color-form combinations. The study showed that a decline in the speed of perception and cognition precedes a decline in linguistic-cognitive abilities in mild to moderate severity of AD [10].

AQT is a good tool for screening early-stages of dementia. it has good validity and reliability [23]. AQT was not related to education but was correlated with age in the Italian older adults [5]. Anderson et al. found that reading time was significantly longer in patients with Dementia with Lewy Bodies (DLB) than in patients with mild Alzheimer’s disease [19]. Another study found that patients with Parkinson’s disease dementia (PDD) had more changes in processing speed than Alzheimer’s disease [24]. In a previous study, the sensitivity and specificity of dementia diagnosis for AQT have been 0.78 and 0.67, respectively, and were higher than for MMSE (0.61) and CDT (0.46) [10]. The test-retest reliability ranges from r = 0.84 to 0.97.

AQT has been tested in many languages and findings indicate that processing speed varies with the syllabic structure of words in a given language or family of language. In English, Danish, and Swedish (Germanic languages), the syllable lengths of the stimulus words are essentially the same and processing-speed times do not differ significantly. For speakers of Italian and Spanish (Romance languages), many of the stimulus words are multisyllabic and the processing speed measures are longer. Therefore, this study aimed to evaluate the test-retest reliability, internal consistency (Cronbach’s alpha), and concurrent validity of A Quick Test of Cognitive Speed (AQT) in screening for mild cognitive impairment and dementia in the Persian language.

## Methods

### Study design

This study was designed to evaluate the psychometric properties of A Quick Test of Cognitive Speed (AQT) in screening for mild cognitive impairment and dementia in the Persian language. AQT is an objective tool and does not require translation; we translated the method of using AQT into Persian and the tool was used in the Persian language. We selected the participants from the psychiatric clinic of the Hazrat Rasoul Akram Hospital and the memory clinic of the School of Behavioral Sciences and Mental Health (Tehran Institute of Psychiatry) of Iran University of Medical Sciences.

### Participants

We determined the sample size based on previous studies [10, 19, 25]. Forty-five participants were entered into the study for each group through convenience sampling and estimating Coefficient Alpha (expected Cronbach’s alpha: 0.7, α: 0.05, the desired width of the confidence interval: 0.3, number of each subscale: 3) [26–28]. The sampling of the dementia group remained unfinished due to the prolongation of the study and problems related to the collection of participants with dementia (COVID-19 Pandemic).

We had three groups: controls (*n* = 45), mild cognitive impairment (MCI) (*n* = 46), and dementia (*n* = 24) based on cognitive status. The sampling method was performed from May 2018 to Feb 2020. Inclusion criteria in the control group include age over 60 years, no complaints of memory, or any other cognitive symptoms, normal cognitive function, no cognitive disorders as approved by two geriatric psychiatrists. The retest-test was assessed for 20 participants from each group (controls, MCI, and dementia). Diagnostic criteria in the MCI group include age over 60 years, confirmation by two geriatric psychiatrists of the presence of MCI (self-report complaint or caregiver report about memory problems, self-report complaint or caregiver report about problems in other cognitive domains, objective memory impairment, objective cognitive disorder, maintain general cognitive function, maintain independence in functional abilities, lack of dementia criteria) [29]. Diagnostic criteria in the dementia group include age over 60 years, major neurocognitive disorder (any type) based on DSM-5 criteria, loss of independence, and approval by two geriatric psychiatrists (Other screening tests e.g. magnetic resonance imaging (MRI) and MMSE) [30]. We only included patients in a study in which both geriatric psychiatrists agreed that the patient had MCI or dementia based on clinical interviews and other evidence. Exclusion criteria included visual problems, make a mistake in the short familiarization trials, and more than 5 incorrect answers in AQT (we did not have incorrect answers in this study). Other causes such as depression, hypothyroidism, and drug side effects that may cause cognitive complaints have been ruled out. We obtained written consent from all study participants if they were able, or from their families after explaining the objectives and methods of study.

### Data collection

We collected data using the AQT and MMSE screening tests. We used a stopwatch to record the time used for completing each of the three AQT tests. The time was recorded in seconds from the beginning of the test to its end. We used the test-retest method to evaluate the reliability of AQT after 1 month for 45 elderly controls.

**Fig. 1:**
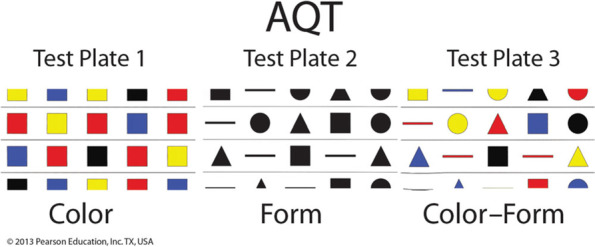
A Quick Test of Cognitive Speed (AQT)

A Quick Test of Cognitive Speed (AQT) is a screening tool for identifying cognitive impairments. It consists of three subtests: Color (C), Form (F), and Color-Form (CF). The time used for rapid automatized naming of the forty visual stimuli in each subtest is measured in seconds. The Color (C) and Form (F) tests measure reaction, retrieval, and response time (perceptual processing) and the Color-Form (CF) combination test assesses visual working memory and active attention [23, 31]. The Color-Form (CF) test is appropriate for examining changes in cognitive function related to neurological or psychiatric disorders and the effectiveness of pharmacological therapies. Naming the combinations activates the bilateral parietotemporal regions and the subcortical region of the brain, including the hippocampus, and examines central executive functions [20, 32]. Administering the three tests in succession takes from 3 to 5 min. The visual stimuli for AQT are presented on three test plates. The first features eight lines of colored squares (black, blue, red, and yellow) and the second eight lines of black forms (circle, line, square, and triangle) that are repeated randomly. The third page consists of eight lines of color and form combinations (Fig. 1). The patient is allowed to use any names to express colors and forms, and time is recorded in seconds according to the test instructions [19]. In a previous study, the sensitivity and specificity of dementia diagnosis for AQT have been 0.78 and 0.67, respectively, and were higher than for MMSE (0.61) and CDT (0.46) [10]. The test-retest reliability ranges from r = 0.84 to 0.96. Naming times were not dependent on sex or formal education after establishing literacy. The cut-off point (in seconds) for average-normal performance was set at one standard deviation above the mean (+ 1SD), for slower than normal between + 1 and + 2 standard deviations, and for abnormal performance at more than + 2 standard deviations for English and Swedish [18, 21].

Mini-Mental State Examination (MMSE): This examination was developed by Folstein et al. in 1975. The score range is 0 to 30. A higher score indicates better cognitive function and a score below 24 is a sign of cognitive impairments. MMSE examines the cognitive state within five areas: time and place orientation, memory, attention, calculation, and language. Its test-retest reliability was reported as 0.89 [33]. The Persian version MMSE psychometric properties showed that the test-retest reliability is 0.78 and its cut-off point is 24, with a sensitivity of 0.90 and a specificity of 0.84, and a score range of 21 to 24 is considered MCI. this tool should be interpreted according to age and education [8].

### Data analysis

Data were analyzed using SPSS v.23 software and a specific statistical plan was used for each of the following objectives. The objectives of this study include:

1. Determining and comparing the mean and standard deviation AQT subtests for the control group, people with MCI, and with dementia. We used descriptive statistics, paired and independent t-tests, and ANOVA test.
2. Investigating the relationship and difference between AQT and demographic variables. We used Pearson correlation, paired and independent t-tests, and ANOVA test.
3. AQT reliability and validity assessment. We used Pearson correlation (r). Cronbach’s alpha for internal consistency.
4. Determining the cut-off point for cognitive disorders screening. We used Receiver Operating Characteristics (ROC curve) for cut-off point, sensitivity, and specificity.

## Results

As shown in Table 1, the number of neurotypical controls was 45 (mean age: 69.24 ± 7.34), with mild cognitive impairment 46 (mean age 74.22 ± 6.21), and with dementia 24 (mean age: 78.54 ± 5.38). There was no significant correlation between age and AQT time for all three subscales in all groups. Table 1 also shows the demographics variables age, gender, marital status, and levels of education. Table 2 shows the time differences between the subscales of AQT based on demographic variables. There is no significant difference in any subscale of AQT with demographic variables in the three groups.

**Table 1:** Demographic variables in three groups of control, with MCI and dementia

		Controls (*n* = 45)		Older patients with MCI (*n* = 46)		Older patients with dementia (*n* = 24)	
		n		n	%	n	%
Age		69.24 ± 7.34		74.22 ± 6.21		78.54 ± 5.38	
Sex	Male	19	42.22	34	73.91	12	50
	Female	26	57.78	12	26.09	12	50
Marital status	Married	32	71.1	26	56.5	9	37.5
	Widow/widower	8	17.8	9	41.3	13	54.2
	Divorced	5	11.1	1	2.2	2	8.3
Education	Illiterate	–	–	11	23.9	9	37.5
	Grade school	4	8.9	24	52.2	9	37.5
	Middle school	6	13.3	2	4.3	2	8.3
	Upper school	11	24.4	7	15.2	4	16.7
	University education	–	–	11	23.9	9	37.5

**Table 2:** AQT naming-time differences (s) based on demographic variables in three groups of control, elderly with MCI and dementia

		Controls (*n* = 45)			Older patients with MCI (*n* = 46)			Older patients with dementia (*n* = 24)		
		Mean ± SD			Mean ± SD			Mean ± SD		
		Color	Form	Color-Form	Color	Form	Color-Form	Color	Form	Color-Form
Groups		38.20 ± 11.03	57.59 ± 13.18	108.08 ± 37.75	38.87 ± 14.21	120.15 ± 45.93	205.25 ± 100.79	84.31 ± 24.17	208.57 ± 64.41	338.67 ± 136.38
*P*- value		*P* < 0.001 Df = 2			*P* < 0.001 Df = 2			*P* < 0.001 Df = 2		
Age		r = 0.09 *P* = 0.55	r = 0.05 *P* = 0.74	r = 0.28 *P* = 0.06	r = 0.01 *P* = 0.98	r = 0.14 *P* = 0.36	r = 0.11 *P* = 0.48	r = − 0.37 *P* = 0.08	r = − 0.41 *P* = 0.06	r = − 0.31 *P* = 0.14
Sex	Male	34.79 ± 8.8	34.74 ± 8.74	78.11 ± 18.9	57.82 ± 14.55	111.29 ± 44.85	198.94 ± 70.88	102.42 ± 41.57	315.25 ± 110.32	315.25 ± 110.32
	Female	40.69 ± 11.96	41.88 ± 16.68	88.85 ± 26.85	56.92 ± 8.65	145.25 ± 40.77	235.83 ± 28.27	113.75 ± 34.38	362.08 ± 159.76	362.08 ± 159.76
*P*- value		*P* = 0.08 Df = 43	*P* = 0.09 Df = 43	*P* = 0.14 Df = 43	*P* = 0.84 Df = 44	*P* = 0.06 Df = 44	*P* = 0.08 Df = 44	*P* = 0.47 Df = 22	*P* = 0.41 Df = 22	*P* = 0.41 Df = 22
Education	Illiterate	–	–	–	63.09 ± 19.75	137.64 ± 47.40	250 ± 64.11	108.33 ± 56.78	221.11 ± 147.87	349.44 ± 102.23
	Grade school	42.5 ± 10.41	45.5 ± 13.1	87.75 ± 8.77	54.29 ± 8.47	114.38 ± 42.75	196.04 ± 62.95	103.89 ± 23.02	200.11 ± 68.71	359.78 ± 185.09
	Middle school	42.17 ± 8.54	51.67 ± 14.22	95.67 ± 20.68	47 ± 2.82	76.50 ± 19.09	135 ± 21.21	102.5 ± 10.61	210 ± 42.42	267.5 ± 60.1
	Upper school	38.73 ± 8.99	35.55 ± 4.46	92.82 ± 23.16	60.86 ± 12.79	112.86 ± 52.58	208.71 ± 54.59	119.75 ± 24.98	178.75 ± 67.87	302.50 ± 123.93
	University education	36.25 ± 12.49	36.08 ± 15.74	77 ± 25.61	66 ± 15.55	162.50 ± 38.89	204 ± 50.91	–	–	–
*P*- value		*P* = 0.55 Df = 44 F = 0.71	*P* = 0.06 Df = 44 F = 2.70	*P* = 0.17 Df = 44 F = 1.73	*P* = 0.2 Df = 45 F = 1.56	*P* = 0.33 Df = 45 F = 1.47	*P* = 0.08 Df = 45 F = 2.24	*P* = 0.92 Df = 23 F = 0.16	*P* = 0.92 Df = 23 F = 0.15	*P* = 0.8 Df = 23 F = 0.33

### Reliability and validity

Table 3 shows the correlation between the test-retest measures for AQT after 1 month for reliability and the concurrent validity of AQT with MMSE. The correlations for all subscales of AQT after 1 month are above 0.80 and significant (*P* < 0.01). Cronbach’s alpha was 0.81.

**Table 3:** Test-retest correlation of AQT and the concurrent validity of AQT with MMSE

	Controls (*n* = 20) (after one month)				Older patients with MCI (*n* = 20) (after one month)				Older patients with dementia (*n* = 20) (after one month)			
*n* = 45	C	F	C-F	MMSE	C	F	C-F	MMSE	C	F	C-F	MMSE
Color	**0.84**^b^	–	–	−0.78^b^	**0.88**^b^	–	–	− 0.70^b^	**0.96**^b^	–	–	− 0.61^a^
Form	–	**0.91**^b^	–	−0.71^b^	–	**0.93**^b^	–	0.65^b^	–	**0.97**^b^	–	−0.52^a^
Color Form	–	–	**0.94**^b^	−0.72^b^	–	–	**0.95**^b^	0.60^b^	–	–	**0.97**^b^	−0.46^a^

^a^Correlation is significant at the 0.05 level (2-tailed)

^b^Correlation is significant at the 0.01 level (2-tailed)

The correlation between AQT and MMSE is also significant, but the correlation is negative because the scores of AQT and MMSE are opposite.

### Sensitivity and specificity

The cut-off points for performance on the AQT subtests were determined with the criterion standard (i.e., diagnosis by two geriatric psychiatrists) for the participants in the control, MCI and dementia groups (Tables 4 and 5). The cut-off point for MCI was 43.50 s for the Color subscale (sensitivity = 0.95, specificity = 0.73, AUC% = 0.88, *P* < 0.001). It was 52 s for the Form subscale (sensitivity = 0.98, specificity = 0.89, AUC% = 0.97, P < 0.001), and 89 s for the Color-Form subscale (sensitivity = 0.98 and specificity = 0.62, AUC% = 0.96, P < 0.001). The cut-off point for the Color subscale elderly with dementia was 62.50 s (sensitivity = 0.87 and specificity = 0.78, AUC% = 0.94, P < 0.001), for Form naming of was 111 s (sensitivity = 0.96 and specificity = 0.46, AUC% = 0.83, P < 0.001), and for Color-Form naming was 197.50 s (sensitivity = 0.91 and specificity = 0.41, AUC% = 0.82, P < 0.001).

**Table 4:** Cut-off points for the AQT subscales in the control group and the elderly with MCI

Color			Form			Color-Form		
Positive if Greater Than or Equal To	Sensitivity	1- Specificity	Positive if Greater Than or Equal To	Sensitivity	1- Specificity	Positive if Greater Than or Equal To	Sensitivity	1- Specificity
33	1.00	0.60	40.50	1.00	0.31	76	1.00	0.63
34	1.00	0.53	42	1.00	0.27	77	1.00	0.60
35	1.00	0.51	44	1.00	0.18	79	1.00	0.53
37	1.00	0.49	46	1.00	0.13	82.50	1.00	0.44
38.50	0.95	0.42	**52**	**0.98**	**0.11**	86.50	1.00	0.42
39.50	0.95	0.38	59	0.89	0.11	**89**	**0.98**	**0.38**
41	0.95	0.33	60	0.89	0.11	90.50	0.94	0.36
42.50	0.95	0.31	61	0.95	0.09	91.50	0.93	0.33
**43.50**	**0.95**	**0.27**	62	0.87	0.09	93	0.93	0.31
44.50	0.94	0.26	66	0.85	0.09	94.50	0.93	0.29
46	0.85	0.24	74	0.83	0.04	95.50	0.93	0.27
AUC% = 0.88			AUC% = 0.97			AUC% = 0.96		
Std. Error = 0.03			Std. Error = 0.01			Std. Error = 0.01		
95% Confidence Interval: 0.81–0.95			95% Confidence Interval: 0.95–1.00			95% Confidence Interval: 0.93–0.99

**Table 5:** Cut-off point AQT subscales in the elderly with MCI and old patients with dementia

Color			Form			Color-Form		
Positive if Greater Than or Equal To	Sensitivity	1- Specificity	Positive if Greater Than or Equal To	Sensitivity	1- Specificity	Positive if Greater Than or Equal To	Sensitivity	1- Specificity
52	1.00	0.63	79	1.00	0.76	146	1.00	0.83
54	1.00	0.61	82.50	1.00	0.70	151.50	1.00	0.80
55.50	1.00	0.43	87.50	1.00	0.67	156	1.00	0.78
57	1.00	0.41	95	1.00	0.63	163.50	1.00	0.76
59	1.00	0.37	102.50	1.00	0.59	169	1.00	0.74
**62.50**	**0.87**	**0.22**	106	0.96	0.59	175	1.00	0.70
67.50	0.87	0.15	**111**	**0.96**	**0.54**	185	0.92	0.65
72.50	0.79	0.15	117.50	0.92	0.54	192.50	0.92	0.63
76	0.75	0.10	122.50	0.83	0.45	**197.50**	**0.91**	**0.59**
78.50	0.75	0.09	127.50	0.71	0.43	205	0.83	0.56
82.50	0.75	0.02	132.50	0.71	0.35	213.50	0.83	0.54
AUC% = 0.94			AUC% = 0.83			AUC% = 0.82		
Std. Error = 0.03			Std. Error = 0.05			Std. Error = 0.05		
95% Confidence Interval: 0.88–0.99			95% Confidence Interval: 0.73–0.93			95% Confidence Interval: 0.71–0.93		

## Discussion

Gender and education do not affect AQT. Nielsen et al. (2006) indicated no difference in time measures between the men and women, but the AQT time measures were shorter for literate than for illiterate old people [22]. In this study, AQT time was faster in higher education but was not statistically significant. In a study that evaluated the relationship between the AQT measures and neuropsychological test scores, no relationship was found between age and AQT naming time [6]. AQT time was not correlated with age in this study. A psychometric study of MMSE scores among the elderly reported significant correlations between MMSE scores and age and education [8]. These results indicate that AQT is less affected by demographic variables.

The findings showed that AQT has suitable levels of reliability and validity for screening mild cognitive impairment (MCI) and dementia among the elderly. Test-retest reliability showed that the correlation of each subscale by itself after 1 month is above 0.84 and Cronbach’s alpha 0.81 shows that the instrument has an acceptable internal consistency. As a comparison, the test-retest reliability of AQT for detecting early-stage dementia in elderly Japanese was found to be 0.88, which was similar to this study [23]. Concurrent validity for AQT was assessed with MMSE, which is a standard questionnaire used to assess cognitive status in its various domains. Our findings showed that all AQT subscale measures had a significant correlation with MMSE (r > − 0.70). Because the scoring for these two tests is opposite in value, less time on AQT indicates better cognitive status, whereas higher scores in MMSE indicate optimal cognitive status. In comparison, Nielsen et al. (2007) assessed the relationship between AQT and MMSE and found significant negative correlations between tests that ranged from − 0.60 to − 0.72 (*P* = 0.01) [6]. Similar findings were obtained in a study of Italian adults by Petrazzuoli et al. [5].

Means and standard deviations for AQT Color, Form, and Color-Form naming times indicate a significant difference between the control, MCI, and dementia groups. Andersson et al. (2007) also found significant differences between naming times for healthy participants and groups with Lewy Bodies’ dementia and AD [19]. Takahashi et al. (2012) found that the mean AQT times for the healthy control group were two times shorter than for the group with MCI and three times shorter than for the group with dementia [23]. These differences can be related to many factors such as characteristics of the Japanese language or different levels of severity of the disease.

We used the criterion gold standard (i.e., diagnosis by two geriatric psychiatrists) to determine the cut-off point based on the ROC curve. The cut-off point for distinguishing healthy elderly from elderly with MCI was 43.50 s for the Color subscale with a sensitivity of 0.95 and specificity of 0.73. It was 52 s with a sensitivity of 0.98 and specificity of 0.89 for the Form subscale and 89 s with a sensitivity of 0.98 and specificity of 0.62 for the Color-Form subscale. Takahashi et al. (2012) also used the MMSE scores to determine the cut-off points. The diagnostic cut-off point for the Color-Form subscale for early-stage dementia was approximately 71 to 72 s with a sensitivity of 0.85 and a specificity of 0.76 [23]. In this study, the cut-off point for the Color subscale for differentiating elderly with MCI with dementia was 62.50 s with a sensitivity of 0.87 and a specificity of 0.78. The cut-off point for Form naming was 111 s with a sensitivity of 0.96 and a specificity of 0.46, and the cut-off point for Color-Form naming was 197.50 s with a sensitivity of 0.91 and a specificity of 0.41. A study with 81 patients of the usefulness of different screening tests for dementia in primary care settings reported that for MMSE the sensitivity was 0.58 and specificity 0.91, for the CDT sensitivity was 0.26 and specificity 0.88, and for AQT the sensitivity was 0.78 and specificity 0.67 [10]. AQT is a suitable tool for primary care centers due to its sensitivity and specificity.

The limitation of this study was the lower number of elderly people with dementia than other groups, but the strength was the distinction between three different cognitive states.

## Conclusion

The findings of this study indicate that A Quick Test of Cognitive Speed (AQT) is a suitable tool for screening the cognitive status of older adults in primary care settings. AQT does not require literacy and is not language-dependent for speakers of dialects and languages belonging to the same family. Therefore, AQT can be used for the initial assessment of the cognitive status of the elderly in all care centers.

## Data Availability

The datasets generated and/or analyzed during the current study are not publicly available as individual privacy could be compromised but are available from the corresponding author on reasonable request.
